# Atypical interference effect of action observation in autism spectrum conditions

**DOI:** 10.1017/S0033291713001335

**Published:** 2013-06-13

**Authors:** J. Cook, D. Swapp, X. Pan, N. Bianchi-Berthouze, S-J. Blakemore

**Affiliations:** 1 UCL Institute of Cognitive Neuroscience, London, UK; 2 Donders Centre for Cognitive Neuroimaging, Radboud Univeristy, Nijmegen, The Netherlands; 3 Department of Psychiatry, University of Cambridge, Herchel Smith Building, Cambridge Biomedical Campus, CambridgeUK; 4 UCL Department of Computer Science, London, UK; 5 UCL Interaction Centre, Malet Place Engineering Building, London, UK

**Keywords:** Autism, biological motion, imitation, interference, mirror neurons

## Abstract

**Background:**

Observing incongruent actions interferes with ongoing action execution. This ‘interference effect’ is larger for observed biological actions than for non-biological actions. The current study used virtual reality to investigate the biological specificity of interference effects of action observation in autism spectrum conditions (ASC).

**Method:**

High-functioning adults with ASC and age- and IQ-matched healthy controls performed horizontal sinusoidal arm movements whilst observing arm movements conducted by a virtual reality agent with either human or robot form, which moved with either biological motion or at a constant velocity. In another condition, participants made the same arm movements while observing a real human. Observed arm movements were either congruent or incongruent with executed arm movements. An interference effect was calculated as the average variance in the incongruent action dimension during observation of incongruent compared with congruent movements.

**Results:**

Control participants exhibited an interference effect when observing real human and virtual human agent incongruent movements but not when observing virtual robot agent movements. Individuals with ASC differed from controls in that they showed no interference effects for real human, virtual human or virtual robot movements.

**Conclusions:**

The current study demonstrates atypical interference effects in ASC.

## Introduction

Observing an incongruent action made by another human interferes with ongoing action execution (Kilner *et al.* 2003, 2007; Chaminade *et al.* 2005; Oztop *et al.* 2005; Bouquet *et al.* 2007; Stanley *et al.* 2007; Gowen *et al.* 2008). For instance, when required to execute an action (e.g. horizontal sinusoidal arm movements) whilst simultaneously observing an incongruent action (e.g. vertical sinusoidal arm movements), individuals' movements are more variable in the direction of the observed action than when observing a congruent action (Kilner *et al.* 2003, 2007; Gowen *et al.* 2008). This ‘interference effect’ is greater for observed real human compared with robot actions (Kilner *et al.* 2003). Furthermore, human sinusoidal arm movements proceed with a characteristic velocity called the minimum-jerk (MJ) velocity profile (Flash & Hogan, 1985). The interference effect is greater for observed typical MJ biological motion (BM) actions than for actions with non-biological constant velocity (CV) motion (Kilner *et al.* 2007; although see Oberman *et al.* 2007 for a counter-example). Thus the mechanism that underpins the interference effect appears to be tuned to BM.

A number of theoretical accounts have suggested that action observation mechanisms may function atypically in autism spectrum conditions (ASC), a developmental disorder characterized by impairments in social interaction, language and communication (APA, 2000). Correspondingly a number of studies have questioned the integrity of BM perception in ASC (Hubert *et al.* 2007; Atkinson, 2009; Murphy *et al.* 2009; Saygin *et al.* 2010). Studies employing point-light display (PLD) stimuli, which depict the whole-body motion of the joints of a person, have reported mixed results. Some studies have shown impairments in ASC in judging whether a PLD moves like a person (Blake *et al.* 2003; Kaiser *et al.* 2010*a*,*b*) and in describing the emotion depicted in the stimulus (Hubert *et al.* 2007; Parron *et al.* 2008). However, other studies have shown typical direction discrimination (Murphy *et al.* 2009; Saygin *et al.* 2010) and identification of action from PLD stimuli in ASC (Hubert *et al.* 2007; Parron *et al.* 2008). A recent study found both preserved and impaired BM processing abilities in a large sample of adolescents with ASC, with deficits in PLD-based BM processing being associated with low intelligence quotient (IQ) (Jones *et al.* 2011).

PLD paradigms typically employ control conditions in which the arrangement of the individual point lights is scrambled, thus impairing global and preserving local motion cues. These studies do not directly investigate perception of the MJ velocity profile (which, as noted above, seems to drive the interference effect). Only one study to date has investigated the perception of MJ BM in ASC (Cook *et al.* 2009). In this study, participants observed animations of human hands that made sinusoidal movements with either BM, CV or linear combinations of these two extremes. Participants were required to pick the less natural animation from two exemplars. Relative to control participants, adults with ASC required a greater difference between the two animations in order to distinguish the less natural. This suggests that individuals with ASC exhibit a reduced sensitivity to the difference between BM and non-BM.

Despite the difficulties with MJ BM perception in ASC, studies have found evidence for typical interference effects. Gowen *et al.* (2008) required participants to execute sinusoidal arm movements whilst observing congruent and incongruent movements in three conditions: real human, dot animation that moved with BM or dot animation that moved at CV. The magnitude of the interference effect did not differ between control adults and adults with ASC in any of these conditions. Interference effects can also be measured in terms of reaction time (e.g. Brass *et al.* 2001) and a number of studies have found typical RT interference effects in ASC (Bird *et al.* 2007; Press *et al.* 2010; Spengler *et al.* 2010). However, as noted above, for typical controls the interference effect is greater for observed human compared with robot actions and, to date, studies of the interference effect in ASC have not employed robot stimuli.

Therapists and teachers are increasingly using robots (Costa *et al.* 2010) and virtual reality (for a review, see Wang & Reid, 2011) to teach social skills to children with ASC. Children with ASC are more likely to exhibit social behaviours such as watching, approaching and touching, when presented with a robot that has robotic rather than human appearance, and when presented with a human wearing a robot costume rather than typical human clothing (Robins *et al.* 2006). Thus robot form may facilitate social behaviour in ASC. In addition, reach-to-grasp actions are facilitated by prior observation of a robot but not a human model in ASC (Pierno *et al.* 2008), whereas control children show the opposite effect. Although this research has been restricted to children, it suggests that individuals with ASC may differ from controls in their reactions to human and robot forms. If this is the case, is it due to form or motion cues or an interaction between form and motion? Existing studies that have measured reactions of individuals with ASC to robots have employed robots that execute humanlike movements (Pierno *et al.* 2008; although note that despite the fact that Robins *et al.* 2006 and Costa *et al.* 2008 state that their robot makes humanlike movements they do not specifically report whether it moves with a MJ velocity profile). Existing studies have not manipulated the kinematics of robot motion and thus have not separated the effect of form and motion on the reactions of individuals with ASC.

Studies of the influence of actor motion on the interference effect in ASC have been restricted to two-dimensional stimuli. In the current study participants executed horizontal sinusoidal arm movements while observing either congruent or incongruent movements conducted by a three-dimensional (3D) virtual reality agent with either human or robot form, the finger-tip of which moved with either BM or CV. Another condition featured the same set-up but with a real human actor. Thus the current study manipulated both actor form and motion to investigate (*a*) whether individuals with ASC show an atypical interference effect difference between human and robot stimuli and (*b*) whether this is the result of form or motion cues or an interaction between form and motion cues.

## Method

### Participants

A total of 15 control participants were recruited from the UCL (University College London) subject pool and 14 participants with ASC were recruited from the Institute of Cognitive Neuroscience autism database. The groups were matched for age [mean (s.d.) control: 37.60 (15.06) years, ASC: 41.07 (14.22) years; *t*_27_ = –0.64, *p* = 0.53], gender (male:female control: 13:2; ASC: 11:3) and full-scale IQ [mean (s.d.) control: 118.93 (8.92), ASC: 114.36 (13.33); *t*_27_ = 1.09, *p* = 0.28], as measured by the Wechsler Abbreviated Scale of Intelligence (Wechsler, 1999).

All participants had normal or corrected-to-normal vision and were screened for exclusion criteria (dyslexia, epilepsy, and any other neurological or psychiatric conditions) prior to taking part. All participants in the ASC group had a diagnosis of autism, Asperger's syndrome or autism spectrum disorder (ASD) from an independent clinician. The Autism Diagnostic Observation Schedule (ADOS; Lord *et al.* 1989) was administered by a researcher trained and experienced in the use of this semi-structured behavioural observation schedule. All participants met the cut-off for a diagnosis of autism spectrum for ADOS total (cut-off score = 7) and for the communication (cut-off score = 2) and reciprocal social interaction (cut-off score = 4) subscales (Table 1). All participants gave informed consent to take part in the study, which was approved by the local ethics committee and performed in accordance with the ethical standards laid down in the 1964 Declaration of Helsinki.

**Table 1.** Participant details^a^

	ASC	Control
Gender, *n*		
Male	8	10
Female	2	2
Mean age, years (s.d.)	40.5 (14.74)	40.0 (15.79)
Mean full-scale IQ (s.d.)	116.60 (15.08)	117.50 (9.48)
Mean performance IQ (s.d.)	110.30 (20.20)	n.a.
Mean verbal IQ (s.d.)	116.00 (11.79)	n.a.
Mean ADOS total (s.d.)	10.30 (3.43)	n.a.
Mean ADOS communication (s.d.)	3.60 (1.07)	n.a.
Mean ADOS reciprocal social interaction (s.d.)	6.70 (2.54)	n.a.

ASC, Autism spectrum conditions; s.d., standard deviation; IQ, intelligence quotient; n.a., not applicable; ADOS, Autism Diagnostic Observation Schedule.

a The ASC and control groups were matched in terms of gender, age and full-scale IQ.

### Design and stimuli

**Fig. 1.**
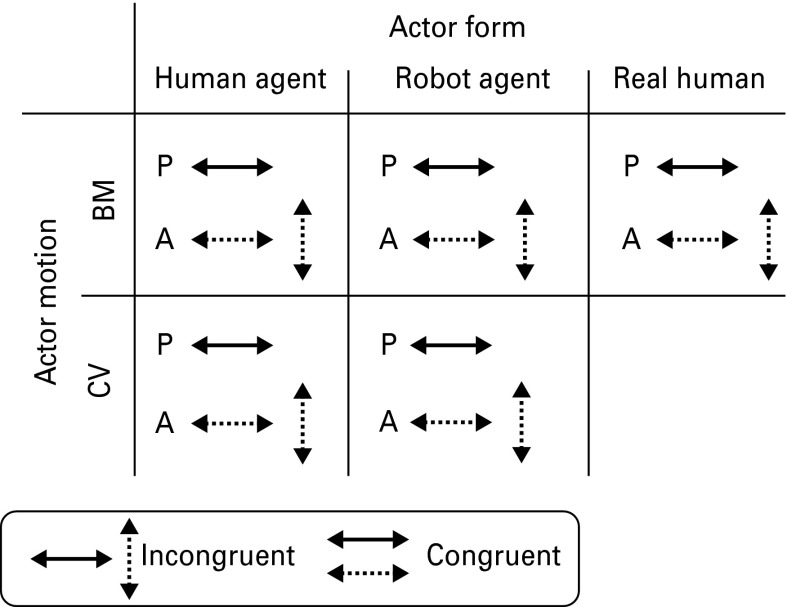
Three different actor forms were employed: human avatar, robot avatar and real human. For the virtual agent conditions two motion types were employed: biological motion (BM) and constant velocity (CV). For 50% of trials in every condition the direction of the movement was congruent with the participant's movement, for 50% of trials the direction was incongruent. In total there were 10 experimental conditions. P, Participant; A, actor.

To compare the influence of both form and motion on executed action in individuals with ASC and controls, we used a 2 (actor form: virtual human agent, virtual robot agent) × 2 (actor motion: BM, CV) × 2 (congruency between participant and actor movement: congruent, incongruent) design for the virtual reality conditions (see Fig. 1). In addition we also investigated the interference effect generated by real human actions in both controls and individuals with ASC.

#### Actor form

There were three different types of actor form: real human, virtual human agent and virtual robot agent. The ‘real human’ was a Caucasian male, aged 31 years. The virtual human agent (online Supplementary Fig. S1A) was represented as a Caucasian male aged around 30 years with similar appearance to the real human. The same skeleton was employed for the robot but all limb segments were replaced with grey cylinders (online Supplementary Fig. S1B). To remove any distracting influence of eye cues the virtual human agent had covered eyes, the virtual robot agent did not feature eyes and the real human had closed eyes. Actors were positioned in the virtual reality theatre such that they appeared to stand 2 m in front of the participant. For each trial only one actor was visible.

#### Actor motion

There were two types of actor motion: BM (for the real human and virtual agent conditions) and CV (for the virtual agent conditions only).

*BM.* The velocity profile of both congruent and incongruent arm movements for the virtual reality human and robot stimuli was created by motion tracking the ‘real human’ actor while he performed sinusoidal vertical and horizontal right arm movements at a rate of 1 Hz. These arm movements were used to animate the right arm of the human and robot virtual agents.

*CV.* CV movements were created by re-sampling the motion-tracked human movement at irregular intervals determined by a linear model. This resulted in sinusoidal movements that preserved the average distance covered (horizontal movements = 807.76 mm; vertical = 977.45 mm), average duration (horizontal = 0.84 s, vertical = 0.86 s), average speed (horizontal = 961.62 mm/s; vertical = 1136.57 mm/s) and trajectory (online Supplementary Fig. S2) of the BM movements. The CV movements differed from the BM movements in that the finger tip of the virtual actor moved across space at a CV rather than following the bell-shaped velocity profile that is characteristic of MJ BM (online Supplementary Fig. S3: Abend *et al.* 1982; Flash & Hogan, 1985).

### Display

The experiment took place in a cave-hybrid immersive virtual reality theatre (Cruz-Neira *et al.* 1992). This consists of three vertical walls and a floor, which make up a continuous projection surface, and onto which 3D computer graphic imagery is projected. The participant wears stereo shutter glasses to enable 3D viewing, as well as a small head-tracking device which allows the projected imagery to be perspective correct for the participant at all times.

### Data recording

Data were recorded using a Vicon motion tracking system (http://www.vicon.com/). Markers that were reflective in infrared were placed in the following positions: finger, wrist, elbow and shoulder of the participant's right arm. The position of each of these sensors was monitored by six infrared cameras at 100 Hz in x, y and z coordinates. There were approximately 100 data samples per movement cycle conducted by each participant.

### Procedure

**Fig. 2.**
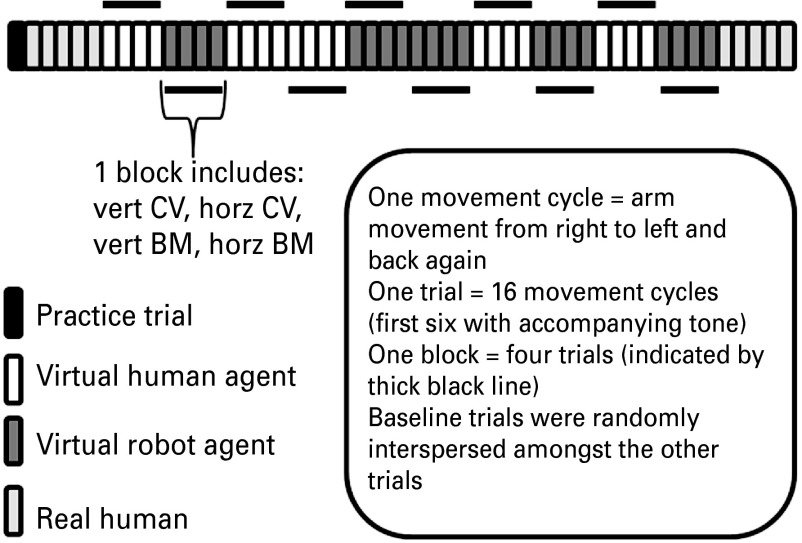
Example trial schedule. Trials were blocked according to the form of the actor. In each block participants saw both congruent and incongruent, biological motion (BM) and constant velocity (CV) trials; thus there were four trials per block. Block order was pseudo-randomized such that no participant saw two or more identical blocks in a row; each participant had a unique pseudo-randomization. Participants conducted five real human trials at the start and five at the end of the experiment. Within the real human condition, congruent and incongruent trials were randomly interleaved. vert, Vertical; horz, horizontal.

Participants stood in the virtual reality theatre and made horizontal sinusoidal movement cycles (where one cycle is a movement from right to left and back again) with their right arm. These arm movements were cued by a sequence of three high-pitched and three low-pitched tones. Participants were instructed to synchronize their movements so that they were at the far right when they heard the high-pitched tone and on the far left when they heard the low-pitched tone. This ensured that participants moved in phase with the actor. Whilst performing the horizontal arm movements, participants watched right arm movements of the virtual human agent, the virtual robot agent, the real human or a blank screen. Arm movements were anatomically matched so as to maintain similarity and comparability with previous investigations of the interference effect (Kilner *et al.* 2003, 2007; Chaminade *et al.* 2005; Oztop *et al.* 2005; Bouquet *et al.* 2007). For each trial the participant completed 16 movement cycles. The first six were accompanied by a tone and the remaining 10 were executed in silence. There were 10 experimental conditions and one baseline condition with five trials for each condition (see Fig. 1). For baseline trials participants were required to conduct horizontal sinusoidal arm movements in front of a blank screen. Data from the baseline trials are not reported in the current paper but have been analysed elsewhere (J. Cook, S-J. Blakemore and C. Press, unpublished observations). Trials were blocked according to the form of the actor. In each block participants saw both congruent and incongruent, BM and CV trials; thus there were four trials per block. Block order was pseudo-randomized such that no participant saw two or more identical blocks in a row; block order was counterbalanced between participants (for an example trial schedule, see Fig. 2). Participants conducted five real human trials at the start and five at the end of the experiment. Within the real human condition, congruent and incongruent trials were randomly interleaved. Prior to recording, the experimenter read standardized instructions and demonstrated the required arm movement. Participants were given one practice trial in which they performed the required movement whilst watching a blank screen.

Participants were given breaks after the first five real human trials, one-third and two-thirds of the way through the virtual agent trials and before the last five real human trials. The entire experiment took 1 h including set-up and breaks.

### Data analysis

Data analysis was based on that employed by Kilner *et al.* (2003, 2007). Data from each participant's finger marker were reconstructed, using Vicon software, in x, y and z dimensions. A total of 10 movement cycles from the middle of each trial were analysed, which (*a*) allowed for the participant's arm movement to align with the visual stimulus and the tone pacemaker and (*b*) removed movements with artifacts associated with the switch between trials. Data were segmented into movement half-cycles from right to left or left to right (for an example of single trial data, see online Supplementary Fig. S4).

#### Interference effect

For each segmented movement cycle the variance in the movement in the vertical plane (the error plane) was calculated. Outlying movement cycles in which the variance was greater than or less than 1.96 s.d. away from the group mean were excluded [the probability that these movements are truly representative of this participant's movement variance is less than 5% (*p*_chance_ < 0.05); online Supplementary Table S1]. The mean variance was calculated across all trials for each condition. For each participant and in each condition, an ‘interference effect’ (an index of the extent to which the observed movement affects the executed movement) was calculated as variance in executed movement half-cycles produced whilst the participant observed a vertical (incongruent) arm movement minus the variance produced whilst the participant observed a horizontal (congruent) arm movement. Data from three participants (two ASC and one control) were excluded because of technical difficulties during data collection, which resulted in error (vertical) plane variance scores greater than 1.96 s.d. away from the mean (*p*_chance_ < 0.05). Data from a further four participants (two ASC and two control) were excluded from the final analysis on the basis that the recorded interference effect was greater than (one ASC and one control) or less than (one ASC and one control) 1.96 s.d. away from the group mean. Data from 10 participants with ASC and 12 control participants were included in the final analysis; these groups did not significantly differ in terms of age (*t*_20_ = –0.08, *p* = 0.94) or full-scale IQ (*t*_20_ = 0.17, *p* = 0.87; Table 1).

Data were analysed with a mixed-model 2 × 2 × 2 × 2 analysis of variance (ANOVA) with factors ‘group’ (ASC, control), ‘actor form’ (virtual human agent, virtual robot agent), ‘actor motion’ (BM, CV) and movement ‘congruency’ (congruent, incongruent); this ANOVA was performed on data from the virtual reality conditions only. Reactions to the real human and the robot were compared using a mixed-model 2 × 2 × 2 ANOVA with factors ‘group’ (ASC, control), ‘actor form’ (real human, virtual robot agent) and ‘congruency’ (congruent, incongruent); since the real human did not move with CV, only the BM trials from the robot condition were included in this analysis.

The number of movement cycles in each condition that each participant completed varied somewhat between participants. To investigate whether the number of movement cycles included in the final analysis varied as a function of any of the factors in our design we conducted these ANOVA models with number of included movement cycles (mean variance – 1.96 × s.d. > variance < mean variance + 1.96 × s.d.) as the dependent variable. There were no main effects of, or interactions between, any of the factors (all *p* > 0.05). Hence, there was no systematic relationship between the number of included movement cycles and group membership or experimental condition.

## Results

All results are *p* < 0.05 Bonferroni corrected for multiple comparisons.

### Interference effect generated by human and virtual robot agents

**Fig. 3.**
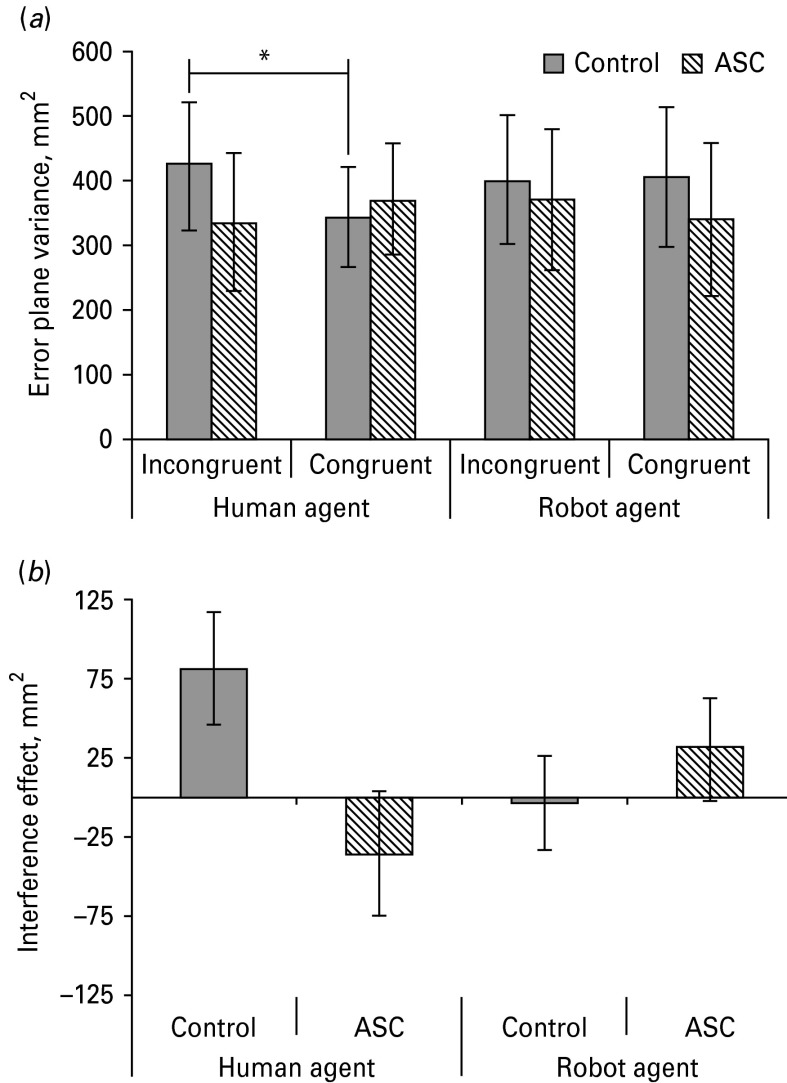
(*a*) Error plane variance. Values are adjusted means, with standard errors represented by vertical bars. * The control group exhibited greater error plane variance when observing a human agent perform incongruent compared with congruent movements (*p* < 0.05). In contrast, for individuals with autism spectrum conditions (ASC), error plane variance was not significantly different for the incongruent and congruent conditions. (*b*) For illustrative purposes, interference effect (incongruent minus congruent error plane variance). Values are adjusted means, with standard errors represented by vertical bars. The control group exhibited a positive interference effect when observing movements conducted by the human agent but not when observing robot agent movements. In contrast, individuals with ASC did not exhibit an interference effect in any condition.

A mixed-model 2 × 2 × 2 × 2 ANOVA with factors ‘group’ (ASC, control), ‘actor form’ (virtual human agent, virtual robot agent), ‘actor motion’ (BM, CV) and movement ‘congruency’ (congruent, incongruent) showed a significant interaction between group × actor form × congruency (*F*_1,20_ = 5.05, *p* = 0.04, *η*_p_^2^ = 0.20). This interaction was also significant if age and (full-scale) IQ were included as covariates (*F*_1,18_ = 4.83, *p* = 0.04, *η*_p_^2^ = 0.20). Simple-effects analyses with age and IQ as covariates demonstrated that, whereas the control group produced significantly more error plane variance when observing incongruent [adjusted mean (s.e.m.) = 423.14 (98.42)] compared with congruent [342.21 (77.50); *F*_1,18_ = 5.12, *p* = 0.04) movements conducted by the virtual human agent, individuals with ASC did not [incongruent adjusted mean (s.e.m.) = 335.03 (107.84), congruent = 370.25 (84.90); *F*_1,18_ = 0.80, *p* = 0.38]. Neither group demonstrated a difference in error plane variance generated whilst observing incongruent and congruent movements performed by the virtual robot agent (all *F*_1,18_ < 1, *p* > 0.3). These results demonstrate that, for the control group, virtual human agent but not virtual robot agent movements produced a significant interference effect, whereas neither virtual human nor robot agent movements produced a significant interference effect for the ASC group (Fig. 3).

The 2 × 2 × 2 × 2 ANOVA also showed a significant actor motion × group interaction (*F*_1,20_ = 6.82, *p* = 0.02, *η*_p_^2^ = 0.25), which was also significant if age and IQ were included as covariates (*F*_1,18_ = 6.78, *p* = 0.02, *η*_p_^2^ = 0.21). Simple-effects analyses with age and IQ as covariates demonstrated that this interaction was driven by a trend towards a difference between the control group and ASC group in error plane variance regardless of congruency: numerically the control group produced more error plane variance when observing BM [adjusted mean (s.e.m.) = 408.47 (95.90)] compared with CV motion [377.15 (91.20)]. This trend did not reach significance (*F*_1,18_ = 4.07, *p* = 0.06) but approached it. However, since this trend did not include the congruency factor it represents only a difference in the general variability of movements rather than a difference in the interference effect. For the ASC group, there was no significant difference (*F*_1,18_ = 2.85, *p* = 0.11) between error plane variance produced when observing BM [339.56 (s.e.m. 105.07)] compared with CV [368.27 (s.e.m. 99.92)] motion.

This analysis revealed no other main effects or interactions (all *p* > 0.05). Note that the lack of a main effect of group demonstrates that, across conditions, both groups exhibited comparable levels of error plane variance whilst performing horizontal arm movements.

### Interference effect generated by real human observation

A mixed-model 2 × 2 × 2 ANOVA with factors ‘group’ (ASC, control), ‘actor form’ (real human, robot) and ‘congruency’ (congruent, incongruent) revealed a significant interaction between group × actor form × congruency (*F*_1,20_ = 4.24, *p* = 0.05). This interaction was also significant when age and IQ were included as covariates (*F*_1,18_ = 4.86, *p* = 0.04, *η*_p_^2^ = 0.21). There were no other main effects or interactions. Simple-effects analyses with age and IQ as covariates revealed that the group × actor form × congruency interaction was driven by a non-significant trend towards a difference between incongruent (adjusted mean (s.e.m.) = 491.04(128.51)] and congruent [416.25 (122.41)] movement observation in the real human condition for the control group [*F*_1,18_ = 2.56, *p* = 0.07 (one-tailed), *η*_p_^2^ = 0.12] but not for the ASC group [incongruent: 292.25 (140.80); congruent: 355.01 (134.11); *F*_1,18_ = 1.48, *p* = 0.24]. Neither group showed a significant difference between incongruent and congruent movement observation in the robot condition [control: incongruent: 411.73 (105.84); congruent: 456.90 (123.53) (*F*_1,18_ = 0.69, *p* = 0.42); ASC: incongruent: 339.13 (115.95); congruent: 329.62 (135.34) (*F*_1,18_ = 0.025, *p* = 0.87); online Supplementary Fig. S5].

## Discussion

The current study used 3D virtual agents to enable the manipulation of actor form (human *v.* robot) and actor motion (BM *v.* CV) whilst keeping all other factors constant. Results showed that control participants exhibited significantly more error plane variance when observing incongruent rather than congruent movements but only if the observed actor was a virtual human agent, not a virtual robot agent. This was not the case for individuals with ASC: for this group error plane variance did not vary as a function of condition. In other words, whereas for controls observing the human agent interfered with their ongoing actions, this was not true for participants with ASC. This result comprises the first demonstration of an atypical interference effect in ASC.

### Interference effect in healthy controls

In line with previous work (Kilner *et al.* 2003), we found that control participants exhibited a greater interference effect for human agent compared with robot agent movements. The lack of a difference in the interference effect between BM and CV conditions for the control group was unexpected. However, as Kilner *et al.* (2007) suggest, this effect may depend on previous experience and expectations of how a stimulus should move. Kilner *et al.* (2007) found no difference in the interference effect generated by BM and CV ball stimuli. The authors argue that, through exposure to computer animations, participants may be equally familiar with CV and BM ball movements. It might therefore be equally possible to simulate the movement of the BM and CV ball and both may create an interference effect. This explanation is of relevance to the current study, which employed computer-animated virtual agents. Our participants may have had previous correlated sensory and motor experience with (for example) computer game virtual agents moving with both BM and CV (e.g. moving a joystick to the right whilst simultaneously observing an on-screen avatar move to the right), which might result in a reduced difference between BM and CV conditions.

### Interference effect in ASC

Whereas control adults exhibited a greater interference effect in response to the human form virtual agent compared with the robot form virtual agent, individuals with ASC did not exhibit this modulatory effect of human form. This result is in line with the finding of Pierno *et al.* (2008) that visuomotor priming was greater for control children relative to children with ASC following observation of human actions. Pierno *et al.* (2008) also demonstrated that visuomotor priming was greater for children with ASC compared with control children following observation of robot actions. Based on these data one might expect a greater robot-action-driven interference effect for individuals with ASC relative to control participants. However, we found no evidence of this. In the current study, the way the ASC group responded to human actions was similar to the way control participants responded to robotic actions. Differences in the predictability and repeatability of the movement stimuli could explain the discrepancy between the current findings and those of Pierno *et al.* (2008): although the reach-to-grasp actions performed by Pierno *et al*.'s robotic actor followed human motion, the duration, average velocity and time-to-grip-aperture was identical in every trial, making the robot movement more predictable than the human movements. Pierno *et al.* suggest that this predictability may promote superior performance in individuals with ASC (Robins *et al.* 2006). More specifically they propose that control participants are more likely to classify and ‘group together’ similar observed movements, perhaps relying on a common template rather than depending on the incoming sensory information. In contrast, individuals with ASC may rely more on their incoming sensory information; thus instead of grouping similar movements, individuals with ASC may have unique perceptual representations of each movement they encounter. In the task employed by Pierno *et al.*, the human movements were statistically more variable than the robot movements. Whereas this variability may have had little effect on the controls if they were relying on a common template, it may have meant that, for individuals with ASC, the human condition was computationally more challenging than the robot condition. Another possible explanation is that individuals with ASC may prefer and thus pay more attention to the predictable robotic motion over the irregular human motion. Typical controls would be unlikely to exhibit this attentional bias towards robotic motion. In the current experiment robot and human movements were matched for duration and average velocity, thus circumventing any potential difference attributable to the variability of movements.

The interaction between group and actor form reported in the current study was driven by a greater interference effect for human compared with robot movements for the control group but not for the ASC group. It should be noted that there were no main effects of group in any of our analyses, suggesting that across the various conditions individuals with ASC and controls exhibited comparable levels of error plane variance. Coupled with the lack of a difference between the groups in the number of trials that had to be discarded from the analysis (online Supplementary Table S1), this suggests that our participants with ASC understood the task instructions and performed the task in a similar way to control participants.

The lack of an interference effect for individuals with ASC contrasts with previous studies (Bird *et al.* 2007; Gowen *et al.* 2008; Press *et al.* 2010, Spengler *et al.* 2010). It is possible that the discrepancy between the current study and previous studies is a result of the different action preparation affordances of the paradigms employed. In previous interference effect paradigms (Bird *et al.* 2007; Gowen *et al.* 2008; Press *et al.* 2010) participants were instructed to make one of two pre-specified actions upon presentation of a cue. In this situation the action not currently executed might be prepared for its imminent execution, hence activating the motor representation of the incongruent action. In such cases, even a weak cortical motor response to action observation may be sufficient for motor activity to reach the motor execution threshold and be expressed as a typical interference effect. In contrast, in the current paradigm the participant was only ever instructed to execute one action type; therefore, action preparation for the incongruent action is unlikely.

It is tempting to interpret the current results as evidence supporting the broken mirror neuron system (MNS) hypothesis of ASC (Ramachandran & Oberman, 2006). The MNS comprises brain areas that are active when an individual observes an action and executes that same action (Iacoboni *et al.* 1999; Catmur *et al.* 2008; Cook, 2012) and is a possible neural substrate for the interference effect (Kilner *et al.* 2003; Blakemore & Frith, 2005; Press *et al.* 2011). In 1991, Rogers & Pennington (1991) suggested that, along with emotion sharing and theory of mind, a deficit in perception-action matching might be a primary difficulty in ASC. It was subsequently suggested that the MNS may function atypically in ASC and that early MNS dysfunction might lead to a cascade of developmental impairments (Williams *et al.* 2001). In support of this a number of studies have demonstrated weaker responses in MNS regions in individuals with ASC compared with controls during movement observation, execution and imitation (Oberman *et al.* 2005; Théoret *et al.* 2005; Dapretto *et al.* 2006; Williams *et al.* 2006; Bernier *et al.* 2007). However, more recent theories have suggested that, rather than the MNS being ‘broken’ in ASC it may be that control over MNS output (e.g. imitation and interference effects) is atypical (Hamilton, 2008; Spengler *et al.* 2010; Kana *et al.* 2011; Cook *et al.* 2012). In other words, these theories propose that the atypicalities lie, not in the MNS *per se* but rather in the inhibition, or facilitation, of mirror responses. Empirical support for this includes a recent study that demonstrated that the social modulation of imitation is atypical in ASC: control participants primed with positive social attitudes showed greater automatic imitation of simple finger movements relative to individuals primed with non-social attitudes. In contrast, there was no difference between pro-socially and non-socially primed individuals with ASC despite them demonstrating the basic imitation effect (Cook & Bird, 2011). Further support includes evidence of difficulties suppressing imitation in ASC (Lhermitte, 1986; Spengler *et al.* 2010), atypical activation of brain areas known to be involved in the control of imitative responses (Spengler *et al.* 2010; Wang *et al.* 2011) and disrupted connectivity between brain regions involved in the control and inhibition of movement and the MNS (Shih *et al.* 2010).

An alternative explanation for the current results could therefore be that the lack of interference effect in ASC is not a direct consequence of a ‘broken MNS’ but rather is a consequence of atypical control over imitation. More precisely, the human form present in the virtual human agent may act as a ‘pro-social prime’ for typical individuals but not for individuals with ASC. For typical controls this priming would result in a release of inhibition of imitative responses, thus resulting in an elevated interference effect relative to the robot condition. If pro-social priming is atypical in ASC, the release of inhibition under the virtual human condition would be absent and the interference effect would remain suppressed.

## Conclusion

Observing arm movements generated by virtual agents with human but not robot form resulted in an interference effect for control participants. In contrast, individuals with ASC showed no interference effect when observing human or robot movements.

## Supplementary material

For supplementary material accompanying this paper visit http://dx.doi.org/10.1017/S0033291713001335.

## Supplementary Material

Supplementary Material Supplementary information supplied by authors.

## References

[ref1] Abend W, Bizzi E, Morasso P (1982). Human arm trajectory formation. Brain 105, 331–348 7082993 10.1093/brain/105.2.331

[ref2] APA (2000). Diagnostic and Statistical Manual of Mental Disorders, 4th edn, text revision. American Psychological Association: Washington, DC

[ref3] Atkinson AP (2009). Impaired recognition of emotions from body movements is associated with elevated motion coherence thresholds in autism spectrum disorders. Neuropsychologia 47, 3023–3029 19500604 10.1016/j.neuropsychologia.2009.05.019

[ref4] Bernier R, Dawson G, Webb S, Murias M (2007). EEG mu rhythm and imitation impairments in individuals with autism spectrum disorder. Brain and Cognition 64, 228–237 17451856 10.1016/j.bandc.2007.03.004 PMC2709976

[ref5] Bird G, Leighton J, Press C, Heyes C (2007). Intact automatic imitation of human and robot actions in autism spectrum disorders. Philosophical Transactions of the Royal Society of London, B: Biological Sciences 274, 3027–3031 10.1098/rspb.2007.1019 PMC2291158 17911053

[ref6] Blake R, Turner LM, Smoski MJ, Pozdol SL, Stone WL (2003). Visual recognition of biological motion is impaired in children with autism. Psychological Science 14, 151–157 12661677 10.1111/1467-9280.01434

[ref7] Blakemore SJ, Frith C (2005). The role of motor contagion in the prediction of action. Neuropsychologia 43, 260–267 15707910 10.1016/j.neuropsychologia.2004.11.012

[ref8] Bouquet CA, Gaurier V, Shipley T, Toussaint L, Blandin Y (2007). Influence of the perception of biological or non-biological motion on movement execution. Journal of Sports Science 25, 519–530 10.1080/02640410600946803 17365539

[ref9] Brass M, Bekkering H, Prinz W (2001). Movement observation affects movement execution in a simple response task. Acta Psychologica (Amsterdam) 106, 3–22 10.1016/s0001-6918(00)00024-x 11256338

[ref10] Catmur C, Gillmeister H, Bird G, Liepelt R, Brass M, Heyes C (2008). Through the looking glass: counter-mirror activation following incompatible sensorimotor learning. European Journal of Neuroscience 28, 1208–1215 18783371 10.1111/j.1460-9568.2008.06419.x

[ref11] Chaminade T, Franklin DW, Oztop E, Cheng G (2005). Motor interference between humans and humanoid robots: effect of biological and artificial motion Proceedings of 2005 4th IEEE International Conference on Development and Learning, pp. 96–101

[ref12] Cook J, Barbalat G, Blakemore SJ (2012). Top-down modulation of the perception of other people in schizophrenia and autism. Frontiers in Human Neuroscience 6, 175 22715325 10.3389/fnhum.2012.00175 PMC3375615

[ref13] Cook J, Bird G (2011). Atypical social modulation of imitation in autism spectrum conditions. Journal of Autism and Developmental Disorders 42, 1045–1051 21833823 10.1007/s10803-011-1341-7 PMC3360861

[ref14] Cook J, Saygin AP, Swain R, Blakemore S (2009). Reduced sensitivity to minimum-jerk biological motion in autism spectrum conditions. Neuropsychologia 47, 3275–3278 19632248 10.1016/j.neuropsychologia.2009.07.010 PMC2779370

[ref15] Cook R (2012). The ontogenetic origins of mirror neurons: evidence from ‘tool-use’ and ‘audiovisual’ mirror neurons. Biology Letters 8, 856–859 22573832 10.1098/rsbl.2012.0192 PMC3440963

[ref16] Costa S, Santos C, Soares F, Ferreira M, Moreira F (2010). Promoting interaction amongst autistic adolescents using robots Conference Proceedings of the IEEE Engineering in Medicine and Biology Society (EMBS), pp. 3856–3859 10.1109/IEMBS.2010.5627905 21097267

[ref17] Cruz-Neira C, Sandin D, DeFanti T, Kenyon R, Hart J (1992). The CAVE: audio visual experience automatic virtual environment. Communications of the ACM 35, 64–72

[ref18] Dapretto M, Davies MS, Pfeifer JH, Scott AA, Sigman M, Bookheimer SY, Iacoboni M (2006). Understanding emotions in others: mirror neuron dysfunction in children with autism spectrum disorders. Nature Neuroscience 9, 28–30 10.1038/nn1611 PMC3713227 16327784

[ref19] Flash T, Hogan N (1985). The coordination of arm movements: an experimentally confirmed mathematical model. Journal of Neuroscience 5, 1688–1703 4020415 10.1523/JNEUROSCI.05-07-01688.1985 PMC6565116

[ref20] Gowen E, Stanley J, Miall RC (2008). Movement interference in autism-spectrum disorder. Neuropsychologia 46, 1060–1068 18096192 10.1016/j.neuropsychologia.2007.11.004 PMC6010145

[ref21] Hamilton A (2008). Emulation and mimicry for social interaction: a theoretical approach to imitation in autism. Quarterly Journal of Experimental Psychology (Colchester) 61, 101–115 10.1080/17470210701508798 18038342

[ref22] Hubert B, Wicker B, Moore DG, Monfardini E, Duverger H, Da Fonséca D, Deruelle C (2007). Brief report: recognition of emotional and non-emotional biological motion in individuals with autistic spectrum disorders. Journal of Autism and Developmental Disorders 37, 1386–1392 17160459 10.1007/s10803-006-0275-y

[ref23] Iacoboni M, Woods R, Brass M, Bekkering H, Mazziotta J, Rizzolatti G (1999). Cortical mechanisms of human imitation. Science 286, 2526–2528 10617472 10.1126/science.286.5449.2526

[ref24] Jones C, Swettenham J, Charman T, Marsden AJS, Tregay J, Baird G, Simonoff E, Happé F (2011). No evidence for a fundamental visual motion processing deficit in adolescents with autism spectrum disorders. Autism Research 4, 347–357 21850664 10.1002/aur.209

[ref25] Kaiser M, Delmolino L, Tanaka J, Shiffrar M (2010 *a*). Comparison of visual sensitivity to human and object motion in autism spectrum disorder. Autism Research 3, 191–195 20533450 10.1002/aur.137

[ref26] Kaiser M, Hudac C, Shultz S, Lee S, Cheung C, Berken A, Deen B, Pitskel NB, Sugrue DR, Voos AC, Saulnier CA, Ventola P, Wolf JM, Klin A, Vander Wyk BC, Pelphrey KA (2010 *b*). Neural signatures of autism. Proceedings of the National Academy of Sciences USA 107, 21223–21228 10.1073/pnas.1010412107 PMC3000300 21078973

[ref27] Kana R, Wadsworth H, Travers B (2011). A systems level analysis of the mirror neuron hypothesis and imitation impairments in autism spectrum disorders. Neuroscience and Biobehavioral Reviews 35, 894–902 20974171 10.1016/j.neubiorev.2010.10.007

[ref28] Kilner J, Hamilton A, Blakemore S (2007). Interference effect of observed human movement on action is due to velocity profile of biological motion. Social Neuroscience 2, 158–166 18633814 10.1080/17470910701428190

[ref29] Kilner J, Paulignan Y, Blakemore S (2003). An interference effect of observed biological movement on action. Current Biology 13, 522–525 12646137 10.1016/s0960-9822(03)00165-9

[ref30] Lhermitte F (1986). Human autonomy and the frontal lobes. Part II: Patient behavior in complex and social situations: the ‘environmental dependency syndrome’. Annals of Neurology 19, 335–343 3707085 10.1002/ana.410190405

[ref31] Lord C, Rutter M, Goode S, Heemsbergen J, Jordan H, Mawhood L, Schopler E (1989). Autism Diagnostic Observation Schedule: a standardized observation of communicative and social behavior. Journal of Autism and Developmental Disorders 19, 185–212 2745388 10.1007/BF02211841

[ref32] Murphy P, Brady N, Fitzgerald M, Troje N (2009). No evidence for impaired perception of biological motion in adults with autistic spectrum disorders. Neuropsychologia 47, 3225–3235 19666038 10.1016/j.neuropsychologia.2009.07.026

[ref33] Oberman L, Hubbard E, McCleery J, Altschuler E, Ramachandran V, Pineda J (2005). EEG evidence for mirror neuron dysfunction in autism spectrum disorders. Cognitive Brain Research 24, 190–198 15993757 10.1016/j.cogbrainres.2005.01.014

[ref34] Oberman L, McCleery J, Ramachandran V, Pineda J (2007). EEG evidence for mirror neuron activity during the observation of human and robot action: towards an analysis of the human qualities of interactive robots. Neurocomputing 70, 2194–2203

[ref35] Oztop E, Franklin DW, Chaminade T, Cheng G (2005). Human–humanoid interaction: is a humanoid robot perceived as a human? International Journal of Humanoid Robotics 2, 537–559

[ref36] Parron C, Da Fonseca D, Santos S, Moore D, Monfardini E, Deruelle C (2008). Recognition of biological motion in children with autistic spectrum disorders. Autism: International Journal of Research and Practice 12, 261–274 10.1177/1362361307089520 18445735

[ref37] Pierno A, Mari M, Lusher D, Castiello U (2008). Robotic movement elicits visuomotor priming in children with autism. Neuropsychologia 46, 448–454 17920641 10.1016/j.neuropsychologia.2007.08.020

[ref38] Press C, Cook J, Blakemore SJ, Kilner J (2011). Dynamic modulation of human motor activity when observing actions. Journal of Neuroscience 31, 2792–2800 21414901 10.1523/JNEUROSCI.1595-10.2011 PMC3398132

[ref39] Press C, Richardson D, Bird G (2010). Intact imitation of emotional facial actions in autism spectrum conditions. Neuropsychologia 48, 3291–3297 20638398 10.1016/j.neuropsychologia.2010.07.012 PMC3221037

[ref40] Ramachandran V, Oberman L (2006). Broken mirrors: a theory of autism. Scientific American 295, 62–69 17076085 10.1038/scientificamerican1106-62

[ref41] Robins B, Dautenhahn K, Dubowsky J (2006). Does appearance matter in the interaction of children with autism with a humanoid robot? Interaction Studies 7, 479–512

[ref42] Rogers SJ, Pennington BF (1991). A theoretical approach to the deficits in infantile autism. Development and Psychopathology 3, 137–162

[ref43] Saygin AP, Cook J, Blakemore SJ (2010). Unaffected perceptual thresholds for biological and non-biological form-from-motion perception in autism spectrum conditions. PLoS ONE 5, e13491 20976151 10.1371/journal.pone.0013491 PMC2956672

[ref44] Shih P, Shen M, Ottl B, Keehn B, Gaffrey M, Müller R (2010). Atypical network connectivity for imitation in autism spectrum disorder. Neuropsychologia 48, 2931–2939 20558187 10.1016/j.neuropsychologia.2010.05.035 PMC3315839

[ref45] Spengler S, Bird G, Brass M (2010). Hyperimitation of actions is related to reduced understanding of others' minds in autism spectrum conditions. Biological Psychiatry 68, 1148–1155 21130224 10.1016/j.biopsych.2010.09.017

[ref46] Stanley J, Gowen E, Miall C (2007). Effects of agency on movement interference during observation of a moving dot stimulus. Journal of Experimental Psychology: Human Perception and Performance 33, 915–926 17683237 10.1037/0096-1523.33.4.915 PMC3073012

[ref47] Théoret H, Halligan E, Kobayashi M, Fregni F, Tager-Flusberg H, Pascual-Leone A (2005). Impaired motor facilitation during action observation in individuals with autism spectrum disorder. Current Biology 15, R84–R85 15694294 10.1016/j.cub.2005.01.022

[ref48] Wang M, Reid D (2011). Virtual reality in pediatric neurorehabilitation: attention deficit hyperactivity disorder, autism and cerebral palsy. Neuroepidemiology 36, 2–18 21088430 10.1159/000320847

[ref49] Wang Y, Ramsey R, Hamilton AF (2011). The control of mimicry by eye contact is mediated by medial prefrontal cortex. Journal of Neuroscience 31, 12001–12010 21849560 10.1523/JNEUROSCI.0845-11.2011 PMC6623188

[ref50] Wechsler D (1999). Wechsler Abbreviated Scale of Intelligence. Psychological Corporation: Harcourt Brace and Company: New York, NY

[ref51] Williams J, Waiter G, Gilchrist A, Perrett D, Murray A, Whiten A (2006). Neural mechanisms of imitation and ‘mirror neuron’ functioning in autistic spectrum disorder. Neuropsychologia 44, 610–621 16140346 10.1016/j.neuropsychologia.2005.06.010

[ref52] Williams J, Whiten A, Suddendorf T, Perrett D (2001). Imitation, mirror neurons and autism. Neuroscience and Biobehavioural Reviews 25, 287–295 10.1016/s0149-7634(01)00014-8 11445135

