# Supplementary material for: Atypical interference effect of action observation in autism spectrum conditions
Source: Psychol Med. 2013 Jun 13;44(4):731–40. doi: 10.1017/S0033291713001335 (PMC3898726; doi:10.1017/S0033291713001335)
Supplement: Supplementary Material — Supplementary information supplied by authors. [file S0033291713001335sup001.doc]

**SUPPLEMENTARY MATERIAL**

**
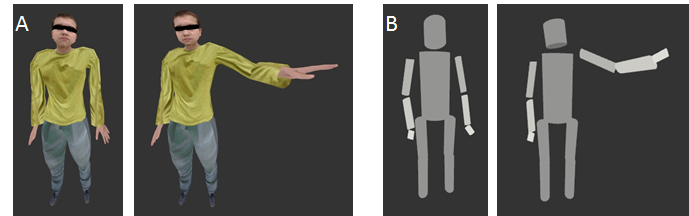
**

**Fig. S1A** The virtual human agent was represented as a Caucasian male aged around 30 years with similar appearance to the real human. **B.** The virtual robot agent was created by replacing the limb segments of the human agent with grey cylinders.


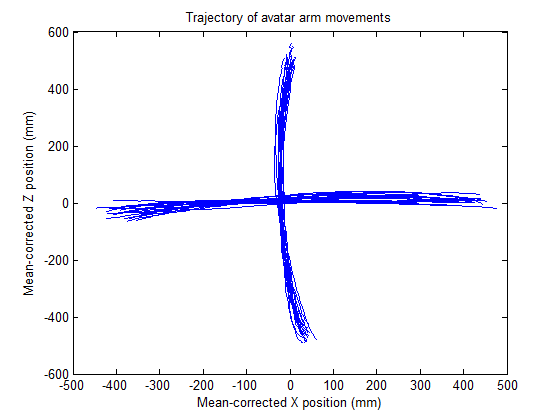


**Fig. S2.** Arm movement trajectories for vertical and horizontal movements. Both biological and constant velocity movements followed the same trajectories.


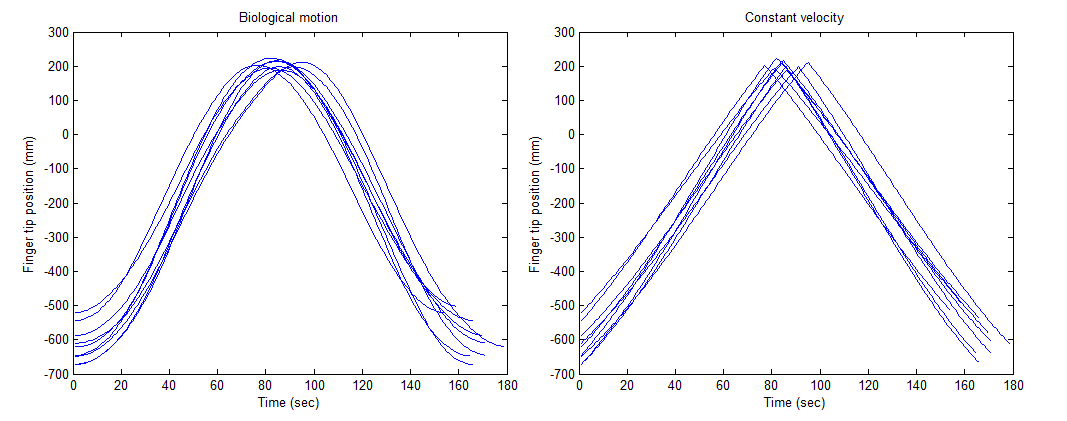


**Fig. S3.** Biological motion and constant velocity movements were matched in terms of average distance covered, average duration, average speed and trajectory but differed in that, for biological movements, the finger-tip followed a bell-shaped velocity profile, whereas for CV movements the finger-tip moved at a constant velocity. A 2x2 ANOVA with factors direction (vertical, horizontal) and velocity (BM, CV) conducted on incongruent variance in the virtual agent movements revealed no main effect of direction (F(1,26) = 3.10, p = 0.09) or velocity (F(1,26) = 1.31, p = 0.26) and no interaction between direction and velocity (F(1,26) = 0.25, p = 0.88). Thus the variability of observed movements did not systematically differ as a function of direction or velocity profile.


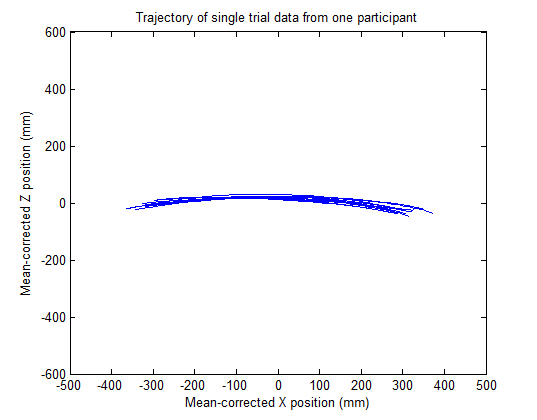


**Fig. S4.** Example arm movement trajectory single trial from an individual participant drawn at random.


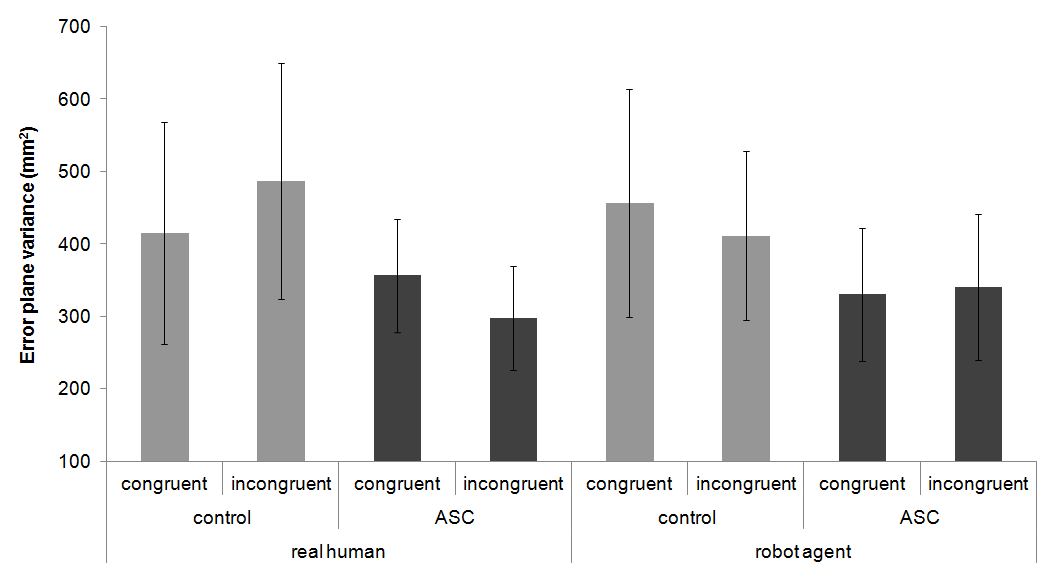


**Fig. S5.** Error plane variance while participants observed sinusoidal arm movements in the congruent or incongruent dimension conducted by either a real human or a robot virtual agent. A 2x2x2 ANOVA with factors Group (ASC, control), Actor Form (real human, virtual robot agent) and Congruency (congruent, incongruent) revealed an interaction between **Group x Actor Form x Congruency** driven by a trend towards a difference between incongruent and congruent movement observation, in the real human condition, for the control group only.

**Table S1.** Included movement cycles by condition and group (the maximum is 50). There was no systematic relationship between the number of movement cycles included in the analysis and the condition or participant group.

| **Form** | **Congruency** | **Motion** | **Control** | | **ASC** | |
| --- | --- | --- | --- | --- | --- | --- |
|  |  |  | **Mean** | **SD** | **Mean** | **SD** |
| Human agent | Incongruent | Biological | 40.20 | 7.26 | 36.9 | 8.5 |
| Human agent | Incongruent | CV | 39.60 | 10.06 | 36.3 | 12.3 |
| Human agent | Congruent | Biological | 41.00 | 7.47 | 39.1 | 7.8 |
| Human agent | Congruent | CV | 40.80 | 9.14 | 39.7 | 8.7 |
| Robot agent | Incongruent | Biological | 42.27 | 6.20 | 38.5 | 9.6 |
| Robot agent | Incongruent | CV | 40.40 | 8.97 | 39.2 | 9.6 |
| Robot agent | Congruent | Biological | 40.07 | 8.90 | 40.7 | 8.2 |
| Robot agent | Congruent | CV | 38.87 | 9.69 | 36.5 | 10.8 |
| Real human | Incongruent | Biological | 36.60 | 13.69 | 43.4 | 5.5 |
| Real human | Congruent | Biological | 40.40 | 8.37 | 43.9 | 5.3 |
